# Frontal lobe function in temporal lobe epilepsy

**DOI:** 10.1016/j.eplepsyres.2011.10.009

**Published:** 2012-01

**Authors:** J. Stretton, P.J. Thompson

**Affiliations:** Department of Clinical and Experimental Epilepsy, Institute of Neurology, University College London, London, UK

**Keywords:** Temporal lobe epilepsy, Cognition, Working memory, Executive function, Neuroimaging

## Abstract

Temporal lobe epilepsy (TLE) is typically associated with long-term memory dysfunction. The frontal lobes support high-level cognition comprising executive skills and working memory that is vital for daily life functioning. Deficits in these functions have been increasingly reported in TLE. Evidence from both the neuropsychological and neuroimaging literature suggests both executive function and working memory are compromised in the presence of TLE. In relation to executive impairment, particular focus has been paid to set shifting as measured by the Wisconsin Card Sorting Task. Other discrete executive functions such as decision-making and theory of mind also appear vulnerable but have received little attention. With regard to working memory, the medial temporal lobe structures appear have a more critical role, but with emerging evidence of hippocampal dependent and independent processes. The relative role of underlying pathology and seizure spread is likely to have considerable bearing upon the cognitive phenotype and trajectory in TLE. The identification of the nature of frontal lobe dysfunction in TLE thus has important clinical implications for prognosis and surgical management. Longitudinal neuropsychological and neuroimaging studies assessing frontal lobe function in TLE patients pre- and postoperatively will improve our understanding further.

## Introduction

Temporal lobe epilepsy (TLE) is associated with significant cognitive impairment. Difficulties in the formation and storage of long-term episodic memories (LTM) have long been recognised as a hallmark of pathological damage to medial temporal lobe structures (MTL). Frontal lobe function has been considered spared in the presence of MTL damage in TLE (Cave and Squire, 1992; Squire et al., 2004), although this assumption has been challenged with emerging evidence for temporal lobe involvement in frontal lobe processes (Ranganath and Blumenfeld, 2005).

The frontal lobes primarily support higher-level cognitive processes, comprising executive skills and working memory (Hanna-Pladdy, 2007; Linden, 2007; Gilbert and Burgess, 2008). Executive functions include vital cognitive activities including decision-making, planning, sustained attention, awareness and insight. Not surprisingly, processing deficiencies can have far ranging effects that impact on educational attainment, employment and social functioning. For instance, executive skills deficits have been associated with poor outcomes on cognitive rehabilitation programmes (Ehlhardt et al., 2008).

**Figure 1:**
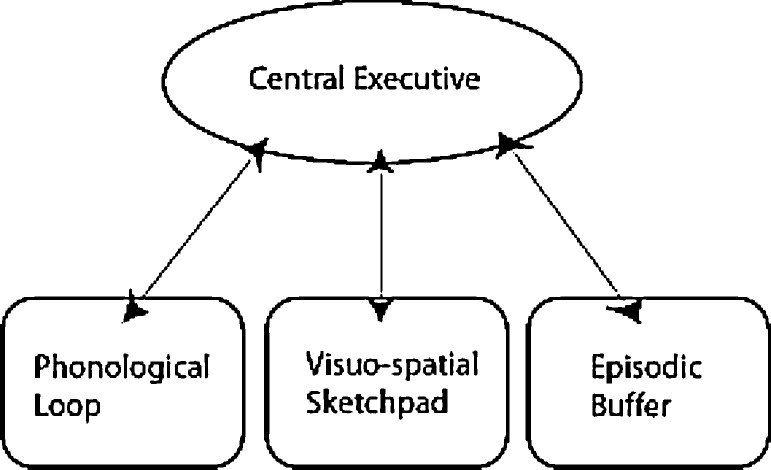
Schematic representation of Baddeley's working memory model (Baddeley, 2000).

Working memory refers to the temporary storage and manipulation of information, and it is an early and key stage in almost all cognitive processing. Impaired function can disrupt subsequent cognition and as a consequence can have a marked impact on even basic everyday activities such as following a conversation or reading a newspaper. Much research has been undertaken on working memory (Baddeley, 2000) and this indicates two subsystems, the phonological loop for the initial processing and storage of verbal information, and the visuospatial sketchpad for early processing of non-verbal information. A third subsystem, the episodic buffer, is responsible for linking and storing information across domains into a multimodal representation, and it is proposed to link the working memory system to the episodic memory system. The working memory subsystems are coordinated by a central executive, responsible for binding information from multiple sources, in order to control and regulate higher cognitive processes (Fig. 1). The presumed bi-directional exchange between these systems indicates a crucial role for fronto-temporal pathways in working memory.

Evidence from psychiatric and neurodegenerative disorders support temporal lobe involvement in classically frontal lobe processes. In schizophrenia, executive dysfunction has been associated with disrupted frontotemporal connectivity (Ragland et al., 2007), while evidence from Alzheimer's Disease and frontotemporal dementia points to caution in the automatic attribution of working memory and executive function failures solely to frontal lobe impairment, suggesting temporoparietal regions are also implicated (Stopford et al., 2011). Frontal lobe function in TLE has received limited attention, with the nature and extent of extratemporal cognitive impairment in TLE remaining poorly understood.

In TLE, whether frontal lobe impairment is a product of critical temporal lobe involvement or is secondary to propagation of epileptic activity from the epileptogenic zone to eloquent cortex responsible for frontal lobe function is subject of current debate (Devinsky, 2005). Identification of the mechanism of impairment holds significant clinical value, for instance in the surgical management of medically refractory TLE patients regarding predictions relating to likely loss or gain of function following anterior temporal lobe resection.

This review will examine the evidence for frontal lobe dysfunction in TLE, focussing on executive functions, working memory, and the potential mechanisms of impairment. A search of Pubmed for original and review articles of adults, in English using a combination of the keywords frontal, temporal, epilep*, working + memory and executive + function was performed. An initial total of 160 papers were identified. Animal research was discounted due to the ongoing debate surrounding the validity of the clinical translation of animal models to human frontal lobe function (Keeler and Robbins, 2011; Penn and Povinelli, 2007). Case reports were also excluded. Relevant citations in the remaining 40 papers were followed up.

## Frontal lobe function in temporal lobe epilepsy

Neuropsychological impairment is an important comorbidity of chronic epilepsy (Thompson and Duncan, 2005). Focal epilepsy syndromes emphasize the link between the primary epileptogenic region and the corresponding cognitive impairment, such as episodic memory in TLE and executive function in frontal lobe epilepsy (FLE) (Elger et al., 2004). In TLE, a pattern of relatively generalised cognitive impairment has been reported which raises the possibility that structural and functional abnormalities may exist outside the bounds of the temporal lobe (Hermann et al., 1997; Oyegbile et al., 2004). This inference has been supported by findings of extratemporal neocortical abnormalities both contralateral and ipsilateral to the side of seizure onset (McDonald et al., 2008; Mueller et al., 2009; Oyegbile et al., 2004). The range of cognitive impairment in TLE has led to attempts to identify cognitive phenotypes of the disorder, with executive functions compromised in a significant subset of the TLE population (Dabbs et al., 2009; Hermann et al., 2007). In one example, Dabbs et al. (2009) applied a cluster analysis to the test scores of 55 chronic TLE patients. Twenty-four percent of the sample showed severe cognitive compromise in memory, executive function and psychomotor speed. Compared to healthy controls and a memory only compromised group, this subset presented with increased abnormalities in total white and grey matter volume, as well as poorer prospective cognitive trajectories.

## Executive functions in temporal lobe epilepsy

The main findings from studies assessing executive function in TLE are summarized in Table 1.

### Neuropsychological studies

Evidence for executive dysfunction in TLE has frequently been investigated using the Wisconsin Card Sorting Task (WCST) (Berg, 1948; Heaton, 1981) or its modified version (Nelson, 1976). Each assesses planning, organisation and the use of environmental feedback to shift cognitive set, all of which are considered classic executive functions. Hermann et al. (1991) examined WCST performance in 64 unilateral TLE patients. Forty four percent of patients exhibited a clinically relevant executive dysfunction. The authors suggested deficits in executive function may be associated with propagation of temporal lobe seizure activity to executive skill relevant areas. In contrast to this hypothesis, Corcoran and Upton (1993) found hippocampal sclerosis (HS), a pathological hallmark of medial TLE, compromised performance on the modified WSCT (MWCST). HS patients completed fewer categories and made more perseverative errors compared to FLE and non-HS TLE groups. Performance on other executive tasks was not compromised. It was argued that the heavy working memory load required to complete the task was responsible for the selective impairment on the MWCST, providing evidence for a dissociation between executive subsystems (Corcoran and Upton, 1993). Similarly, Strauss et al. (1993) assessed 77 TLE patients with the WCST. Set-shifting ability was most impaired by the presence of left temporal lobe dysfunction, but only if damage occurred before the age of one year old. Deficits in right TLE set-shifting ability was less severe, but occurred independently of age of onset. While suggestive of temporal lobe involvement in executive tasks, the impact of seizure frequency and type were not assessed, limiting the interpretation regarding the mechanism of impairment, Subsequent studies have also found performance on the WCST to be compromised in TLE (Drake et al., 2000; Hermann et al., 2007; Horner et al., 1996; Kim et al., 2007; Oyegbile et al., 2004) and specifically those with HS (Allegri et al., 1999; Garcia Espinosa et al., 2010; Giovagnoli, 2001; Oddo et al., 2003). In a comprehensive study, Giovagnoli (2001) investigated the contribution of the hippocampus to performance on the MWCST. The performance of TLE patients with left HS was significantly impaired. There was also a trend for left TLE patients without HS to perform poorly. The authors argued that HS patients were compromised in their ability to form associations and register new information, two processes that are critical for the successful completion of the task. Interestingly, although HS patients performed poorly on a measure of working memory, there was no correlation between working memory and MWCST scores, indicating that working memory disruption may not be related to impaired executive performance providing tentative evidence for a dissociation of the effect of HS on executive subsystems.

There is limited research using other measures of executive functions in TLE. Labudda et al. (2009) assessed decision-making in 20 TLE patients using the Iowa Gambling Task (IGT) (Bechara et al., 1994). This test is designed to assess how feedback affects the decision-making process. Subjects are required to select a card (typically with monetary value) from one of four decks, two decks provide short-term gain and long-term loss (disadvantageous decision), and two provide short-term loss but long-term gain (advantageous decision). Subjects are assessed on their ability to utilise the immediate feedback from each deck in order to make greater advantageous than disadvantageous decisions. Compared to controls, TLE patients were significantly impaired in their decision-making, and those with a preference for disadvantageous decisions performed less well on other tests of executive function. The authors argued that the disturbance in this type of decision-making was due to disrupted feedback processing caused by damage to the amygdala. However, it was noted that those patients with selective HS and no amygdala disturbance also exhibited a preference for disadvantageous decisions (Labudda et al., 2009).

Schacher et al. (2006) investigated advanced theory of mind capacity in TLE through the detection of social faux pas, a sensitive indicator of higher-order deficits. The task requires the detection of social faux-pas (i.e. where someone makes a social blunder) from short scenarios. The performance of 27 medial TLE (MTLE) patients, was compared to 27 demographically matched non-medial TLE patients and 12 healthy controls. The MTLE group performed less well than both non-medial TLE patients and healthy controls in detecting social faux pas. There was no significant difference in performance between non-medial TLE and controls. Across both epilepsy groups, performance did not correlate with epilepsy related variables such as age at seizure onset, or duration of epilepsy. The authors suggest that medial temporal lobe damage, particularly the involving the amygdala was the specific cause of the deficit (Schacher et al., 2006).

While these studies provide evidence for executive dysfunction in TLE, there are also contrary findings. McDonald et al. (2005) administered the Trail Making Test, a measure of mental flexibility, to frontal lobe epilepsy patients (FLE), TLE patients and healthy controls. FLE patients showed significant impairment in both speed and accuracy compared to TLE and controls in the more cognitively demanding set-switching condition. On all other measures, TLE patients performed equal to controls. In addition, a small but important study investigated cognitively based daily living tasks in FLE and TLE groups (Cahn-Weiner et al., 2009). These tasks were either memory based or executive skills based. Neither FLE nor TLE groups showed impairment on the executive based daily living tasks. Despite the potential lack of sensitivity of the tasks employed, this study shows that executive functions in daily life may not be affected by focal epilepsy.

### Neuroimaging studies

Few neuroimaging studies have examined the relationship between executive function and TLE. Jokeit et al. (1997) used FDG-PET to show extratemporal hypometabolsim in TLE was related to reduced executive functioning. A neuropsychological test battery tapping executive function was administered to 96 TLE patients within 3 months of scanning. Asymmetric prefrontal hypometabolsim was observed in 26% of TLE patients. This asymmetry had a main effect on the ten measures of frontal lobe function, with a larger asymmetry related to poorer performance. More recently, Takaya et al. (2006) showed TLE patients experiencing frequent seizures were more impaired in set-shifting (measured by the modified WCST and Trail Making Test) than those experiencing rare seizures. This reduced performance correlated with prefrontal hypometabolsim as measured by FDG-PET, providing support for seizure propagation as a mechanism of impairment.

Wang et al. (2007) used diffusion-tensor imaging to examine the relationship between executive function and white matter integrity in TLE. Patients performed significantly less well across all measures of executive function compared to controls, including the Trail-Making, Digit Span and the Stroop Tests. In addition, the fractional anisotropy (FA) values of the thalamus and posterior limb of the left internal capsule were reduced compared to controls. Correlational analysis revealed the FA of this latter area to be positively correlated with Trail-Making performance indicators. The authors concluded that patients with TLE and executive dysfunction show damage to areas other than the frontal lobes, thus areas beyond the frontal lobes may be involved in executive function (Wang et al., 2007). One further study has used quantitative MRI to investigate executive function in TLE (Keller et al., 2009). Relative to 30 controls, 43 patients with unilateral TLE had volume atrophy of the ipsilateral hippocampus and bilateral prefrontal cortex (PFC). Executive function was positively correlated to left dorsal PFC and left hippocampus volumes. The authors suggested extended damage to brain regions remote from the epileptic zone may be responsible for executive deficits observed in TLE. Both of the above studies are important in highlighting evidence for additional structural damage outside the temporal lobes that correlates with executive ability in TLE. Whether this is an effect of seizure propagation or more widespread pathology in TLE requires further attention.

## Working memory in temporal lobe epilepsy

The main findings from studies assessing working memory in TLE are summarized in Table 2.

### Neuropsychological studies

Recent models of working memory highlight the role of the ‘episodic buffer’, linking working memory to long-term memory systems (LTM; Baddeley, 2000). Accordingly, it is reasonable to argue that the pathological effect of TLE on one process (i.e. LTM) may affect the other i.e. working memory. Several neuropsychological studies provide evidence of working memory dysfunction in TLE (Abrahams et al., 1999; Black et al., 2010; Duzel et al., 1996; Owen et al., 1996; Wagner et al., 2009). Duzel and colleagues (1996) induced changes in verbal working memory performance in patients with left TLE using transcranial magnetic stimulation over the temporal lobe. The authors argued that the phonological loop, responsible for the short-term storage of verbal information, has a functionally and anatomically multimodular structure including both frontal and temporal areas. In addition, Abrahams et al. (1999) found patients with right hippocampal damage were impaired on a spatial working memory task, and that hippocampal and parahippocampal gyrus volume negatively correlated with the number of spatial memory errors These findings support a specialized role for the right hippocampus in spatial working memory.

In the only study to look specifically at material-specific lateralization of working memory in TLE, Wagner and colleagues (2009) studied a group of 96 patients with unilateral MTL damage (24 pre-operative and 72 post-operative) on matched verbal and non-verbal supraspan tasks. For each subject, a supraspan set size score and error rate was recorded for each material type. Compared to healthy controls, both left and right TLE groups showed reduced span sizes for both material types. There were no group differences between pre- and postoperative patients but there was a significant interaction between material type and side of pathology. Right TLE patients had a smaller visuospatial span, but not verbal span, compared to left TLE patients. The left TLE group made significantly more errors on the verbal span task than on the visuospatial task when compared to controls and right TLE patients. These results were taken to argue for material-specific lateralization of working memory dysfunction arising from unilateral medial temporal lobe damage (Wagner et al., 2009). The inclusion of both pre- and postoperative TLE in one group confounds the interpretation of results. The seizure propagation hypothesis could have been investigated by comparing the performance of the seizure-free versus the non-seizure-free patients in the postoperative sample. The study highlights again the need for longitudinal data to identify the contribution of the temporal lobes to processes of working memory.

More recently, Black et al. (2010) investigated the effect of the duration of epilepsy and lifetime seizure load on frontal lobe function in 207 TLE patients compared to 216 patients with psychogenic non-epileptic seizures. Previous research suggested that as duration of epilepsy and the number of complex partial seizures increase, executive functions decline (Thompson and Duncan, 2005). The authors derived an impairment index for each subject based on working memory capacity and executive function performance. Multivariate regression analyses revealed that age at onset was the strongest significant predictor of working memory and executive function, with earlier onset predictive of poorer performance. In addition, greater lifetime seizure load was also related to a reduced executive function. The relationship between clinical variables and frontal lobe function in TLE requires further research.

When directly assessing the role of the medial temporal lobes in working memory, there is limited evidence to suggest that temporal lobe structures are not involved. Cowey and Green (1996) investigated the effect of hippocampal sclerosis on working memory. Twelve HS patients were compared to 12 FLE and 12 healthy controls. The working memory tasks were designed to assess the central executive component of Baddeley's working memory model (Fig. 1). Each subject performed two separate short-term memory tasks simultaneously, placing increased demand on the division of attention. The TLE group performed at the same level as controls. As the authors implied, the measure of working memory they used may not have been sensitive to TLE. They were specifically looking for the neural correlate of the central executive, a component of working memory that may not be effected by TLE (Cowey and Green, 1996).

### Neuroimaging studies

The neuroanatomical basis of working memory is often investigated with variants of the ‘*n*-back’ task (Gevins and Cutillo, 1993). Typically, this requires the monitoring of a series of stimuli, responding whenever a stimulus is presented that is the same as the one presented *n* trials previously (where *n* = 1, 2, 3, etc.). This places great demands on working memory, requiring the on-line monitoring, continuous updating and manipulation of remembered information. A recent meta-analysis of 24 functional MRI (fMRI) data sets for variants of the *n*-back paradigm found consistent activation of frontal and parietal cortical regions (Owen et al., 2005). The authors also found evidence for sub-regional material-specific hemispheric lateralization for working memory processes. Verbal compared to nonverbal identity monitoring was associated with increased activation in left ventrolateral prefrontal cortex, whereas nonverbal location monitoring relative to nonverbal identity monitoring was associated with enhanced activation in right dorsolateral prefrontal and posterior parietal cortex (Owen et al., 2005). Increased MTL activation was not identified. Although this may indicate no involvement, it could be that the effect of working memory load was not adequately represented in the analyses. More recent imaging studies have shown MTL involvement in working memory when multiple items are maintained and contrasted with single item storage (Axmacher et al., 2007, 2009; Campo et al., 2005, 2009). An alternative explanation could be the lack of distinction between novel and familiar stimuli in the *n*-back tasks described. Previous research has shown the MTL specifically contributes to the short-term maintenance of information that has no prior representation in the brain rather than to familiar stimuli (Ranganath and D’Esposito, 2001; Stern et al., 2001; Zarahn et al., 2005).

Few neuroimaging studies have examined working memory in TLE. Techniques including event-related potentials (ERP) (Grippo et al., 1996), intracranial depth electrodes (icEEG) (Axmacher et al., 2007, 2008; Krauss et al., 1997) and magnetoencephalography (MEG) (Campo et al., 2009; Cashdollar et al., 2009) have all been utilised. Early studies indicated TLE patients with memory impairment show reduced performance as working memory load increases on a digit-span task accompanied with attenuated ERP response (Grippo et al., 1996). Additionally, mesial temporal spikes have been shown to interfere with both verbal and non-verbal working memory processes, with left hippocampal spikes causing disruption across both domains (Krauss et al., 1997). Recently, MEG has been used to track the time course of MTL activation in TLE patients when performing a verbal working memory task (Campo et al., 2009). Compared to controls, 9 patients with left hippocampal sclerosis showed significantly reduced performance. MEG recordings during the encoding phase indicated an ipsilateral decrease and a contralateral increase of MTL activity in TLE compared to controls.

Axmacher and colleagues (Axmacher et al., 2007, 2008, 2009) have shown neural activity in the MTL mediates working memory processes. TLE patients (9 right, 2 left) performed a visuospatial working memory task while undergoing icEEG recordings (Axmacher et al., 2007). The paradigm assessed working memory loads requiring the maintenance of one, two or four neutral faces. Following a brief (3 s) pause, a probe face would appear, requiring the subject to respond as to whether they had seen the face previously. Twenty-three healthy controls performed the same task with fMRI. The patients were less accurate with increasing memory load compared to controls. When the working memory load was low (single face), there was a sustained decrease of activity in the hippocampus and rhinal cortex compared to baseline. As the load increased there was an increase in hippocampal activity. These results were reinforced by the analysis of the encoding phase in the 4-face trials; with a stepwise increase in hippocampal activity following the presentation of each additional face. Furthermore, gamma-band activity was in keeping with the direct current changes observed, with a sustained decrease for single item maintenance and an increase as load increased. The fMRI results for the control group confirmed the involvement of the MTL in working memory was not due to a disease process or perceptual changes. In addition to the typical working memory frontal and parietal network activations, the left hippocampus showed an increased activation with increasing memory load that paralleled the icEEG findings (Axmacher et al., 2007). It is of note the stimuli were non-verbal (neutral faces) and the fMRI result does not support the traditional material-specific hypotheses of MTL memory function.

A recent fMRI study examined working memory performance in 36 individuals with cryptogenic focal epilepsy; 10 temporal, 13 frontotemporal and 13 frontal foci based on EEG and seizure semiology (Vlooswijk et al., 2011). Compared to controls, patients were impaired on all measures of working memory. Reduced connectivity in a prefrontal network comprising the anterior cingulate cortex, middle and inferior frontal gyri was associated with performance on a measure sensitive to the central executive component of working memory. However, there was no comparison of prefrontal network integrity between patients with temporal and extratemporal foci, limiting interpretation of the results in the context of seizure propagation. Nonetheless, the authors conclude that in the absence of symptomatic lesions, seizure activity with both temporal and extratemporal origin may disrupt prefrontal network integrity associated with working memory (Vlooswijk et al., 2011).

## Postoperative change

An optimal experimental design for studying the mechanism underlying the disruption to frontal lobe function in TLE is to assess for post-surgical change. Anterior temporal lobe resection (ATLR) is with a well-established treatment for medically intractable temporal lobe epilepsy. If medial temporal lobe structures are critical for executive functioning, then preoperative impairments would be expected and these would be maintained or exacerbated by surgery. If however the spread of epileptogenic activity to frontal regions is causing executive dysfunction, then removal of the primary epileptogenic zone should result in a gain of function. Table 3 summarizes the main findings of the studies that have examined frontal lobe function before and after temporal lobe surgery.

Hermann et al. (1988) examined WCST performance in 35 TLE patients compared to a small group of 6 primary generalised epilepsy (PGE) patients. Fifty seven percent of the TLE group were impaired, with an increased perseverative error rate, compared to only 17% of the PGE group. A subset of the TLE group was retested 6 months following temporal lobe resection. These patients produced fewer perseverative responses, indicating a gain in function following the surgical removal of the primary epileptogenic zone. Frisk and Milner (1990) looked at working memory before and after unilateral temporal lobe resection. Working memory capacity was assessed preoperatively and 3–5 days and two weeks postoperatively. While there was a transient decrease of capacity 3–5 days following surgery, at 2 weeks performance returned to preoperative levels. The transient decrease was attributed to overall language disturbance commonly observed following left ATLR. There was, however, no comment on whether the TLE group were impaired at baseline compared to controls, making interpretation difficult. Size of hippocampal resection had no significant effect on performance. No other clinical outcome measures of surgery (e.g. seizure frequency) were reported, again confounding interpretation of the effect of temporal lobe surgery on working memory. In addition, neuropsychological assessment 2 weeks after major resective surgery may be a weak indicator of cognitive outcome, and longer follow up to show stability of their findings would have been instructive (Frisk and Milner, 1990).

Trenerry and Jack (1994) investigated executive function before and 4–5 months post-operatively in 68 TLE patients using the WCST. Preoperative scores indicated executive dysfunction in 42–57% of the sample. Both left and right TLE patients showed no significant change across all WCST performance measures, however, there was a trend for fewer perseverative responses following resection. Performance did not correlate with seizure onset or hippocampal volume (Trenerry and Jack, 1994). The authors argued against a significant role for the hippocampus in WCST performance, yet acknowledged that performance may be related to the propagation of seizure activity. In a subsequent study, Hermann and Seidenberg (1995) again found WCST performance improved in 74 TLE patients 6 months after temporal lobe surgery, suggesting gain of function following a reduction/cessation of seizures.

Martin et al. (1999) assessed dual pathology patients with left temporal lobe developmental malformations with concurrent left medial temporal sclerosis (LMTS) and patients with LMTS only across several cognitive measures. The measure of executive function included the number of perseverative errors on the WCST, and Trail-Making Test scores. Patients performance was within the normal range on both of these tasks, and no changes were found postoperatively (Martin et al., 1999). This could be interpreted as prior functional reorganisation of temporal lobe related executive function, or that the temporal lobe is not relevant to this performance measure. A complementary study investigated executive function more comprehensively (Martin et al., 2000a). The WCST, a Trail-Making Test and a verbal fluency task was administered to 174 TLE patients before and after anterior temporal lobe resection. Again, executive functions as measured by the WCST and Trail-Making Test were unchanged following surgery but verbal fluency performance improved. The authors argued for a selective normalization of executive function (verbal fluency) following anterior temporal lobe surgery (Martin et al., 2000a). In a further report focussing on the WCST, Martin et al. (2000b) assessed 89 patients with TLE, of which 72 went on to have anterior temporal lobe resection. Regression analysis of the effect several clinical variables on preoperative performance proved non-significant. Patients who were seizure free following surgery did not exhibit better WCST performance than those who continued to experience seizures. The results argue against a role of structural MTL abnormalities or seizure propagation in set-shifting ability (Martin et al., 2000b).

Kim et al. (2007) investigated WCST performance pre and postoperatively in a group of 85 medial TLE patients. At baseline, 56% of medial TLE patients had a sorting impairment, with 30% of those being severe. Correlational analysis showed preoperative sorting ability was negatively correlated with postoperative change i.e. those with greater preoperative scores deteriorated more than those with weaker preoperative scores. The authors suggested that sorting ability in TLE patients cannot be explained by hippocampal damage alone, and that seizure propagation to frontal regions may be predominantly responsible (Kim et al., 2007).

Functional neuroimaging studies have been under-utilised to date and these are likely to help in identifying the underlying neural correlates of working memory and executive functions in TLE. To date, there are no functional magnetic resonance imaging studies specifically investigating frontal lobe function in lesional TLE. The identification of these correlates will aid our understanding of the cognitive phenotypes of TLE.

## Conclusions and future directions

Evidence from both the neuropsychological and neuroimaging literature suggests both executive function and working memory can be compromised in the presence of TLE. In relation to executive impairment, particular emphasis has been paid to set shifting as measured by the WCST. Other functions such as decision-making and theory of mind appear vulnerable, but as this review demonstrates these have received little attention. Study samples are often small or suffer from heterogeneity regarding pathology for example by combing pre- and post-surgical cases.

Despite a limited evidence base, the cause of executive skills weakness seems to be the propagation of seizure activity to executive skills dependant regions in the frontal lobes. With regard to working memory, the evidence more consistently supports a direct role of the temporal lobe in the encoding and maintenance of working memory but with emerging evidence of hippocampal-dependent and hippocampal independent processes. The cognitive phenotype and trajectory in TLE will likely vary depending on the underlying mechanism and this has clinical relevance and will be important to establish further.

In the research reviewed little consideration has been given to the role of anti-epileptic medication and its impact on frontal lobe function. For example, there is considerable evidence that topiramate can negatively impact on working memory (Kim et al., 2006; Smith et al., 2006). This deserves attention and could be explored by reassessing executive functions following drug discontinuation in seizure-free surgical patients.

Longitudinal neuropsychological and functional neuroimaging studies assessing executive skills and working memory pre- and post temporal lobe resections hold promise in elucidating the nature and mechanisms underlying frontal lobe dysfunction in TLE.

## Figures and Tables

**Table 1:** Studies of executive function in TLE.

Author (yr)	*n*. (group)	Executive assessment	Results	TLE effecting frontal lobe function?
Hermann et al. (1991)	64 (TLE)	WCST	44% exhibited clinically relevant executive dysfunction	Yes
Corcoran and Upton (1993)	16 (HS)	MWCST, VF	HS reduced performance on MWCST	Yes
	13 (TLEo)	Stroop		
	18 (FLE)			
Strauss et al. (1993)	77 (TLE)	WCST	Poor performance related to left sided pathology and early age of onset	Yes
Horner et al. (1996)	38 (TLE)	WCST	50% TLE patients showed clinical executive dysfunction as measured by perseverative responses	Yes
Jokeit et al. (1997)	96 (TLE)	‘Frontal’ battery including TMT and digit span	26% patients showed reduced prefrontal hypometabolsim, significantly effecting performance on frontal measures	Yes
Allegri et al. (1999)	50 (MTLE)	WCST, VF, TMTb	MTLE (particularly HS) showed reduced executive performance across all tasks	
	20 (HC)			Yes
	16 (TLE)	WCST	75% TLE patients showed	
Drake et al. (2000)	12 (PGE)		Reduced performance compared to 12% PGE	Yes
Giovagnoli (2001)	112 (TLE)	MWCST	Left FLE and Left HS sig. impaired on MWCST	
	53 (FLE)			Yes
	36 (HC)			
Oddo et al. (2003)	71 (HS)	WCST, Stroop, TMTb	25% showed impaired WCST performance	Yes
McDonald et al. (2005)	23 (FLE)	TMTb, D-KEFS	TLE performance equal to controls	No
	20 (TLE)			
	23 (HC)			
Schacher et al. (2006)	27 (MTLE)	Faux-pas test	MTLE impaired in recognising social faux-pas	Yes
	27 (TLEo)			
	12 (HC)			
Takaya et al. (2006)	21 (MTLE)	MWCST, TMT	Patients with frequent seizures more impaired in set-shifting; related to prefrontal hypometabolism	Yes
Hermann et al. (2007)	96 (TLE)	WCST, Stroop, TMTb,	Cluster analysis revealed 29% TLE belonged to a memory, executive and speed impaired group	Yes
	82 (HC)			
	32 (TLE)	VF, Stroop	TLE reduced performance on all measures	
Wang et al. (2007)	42 (HC)			Yes
Cahn-Weiner et al. (2009)	29 (TLE)	Executive daily living test	Both groups within normal limits	No
	9 (FLE)			
Keller et al. (2009)	43 (TLE)	Stroop, VF	Volume atrophy of dorsal prefrontal cortex related to poorer executive performance	Yes
	30 (HC)			
Labudda et al. (2009)	20 (TLE)	IGT, Game of Dice	TLE, in particular HS patient show reduced performance in IGT	Yes
	20 (HC)			
Black et al. (2010)	207 (TLE)	WCST, Stroop, VF	Early age of TLE onset predicts poorer outcome for each measure	Yes
	216 (PES)			
Garcia Espinosa et al. (2010)	42 (TLE)	WCST	Reduced WCST performance related to increased depressive symptoms	Yes

HS: hippocampal sclerosis; TLE: temporal lobe epilepsy; MTLE: mesial temporal lobe epilepsy; TLEo: temporal lobe epilepsy without hippocampal sclerosis; FLE: frontal lobe epilepsy; cTLE: children with temporal lobe epilepsy; PGE: primary generalised epilepsy; HC: healthy controls; WCST: Wisconsin card sorting task; MWCST: modified WCST; VF: verbal fluency; IGT: Iowa gambling task; TMTb: Trail-Making-Test part B; D-KEFS: Delis–Kaplan executive function system test.

**Table 2:** Studies of working memory in TLE.

Author (yr)	*n*. (group)	Working memory assessment	Results	TLE effecting frontal lobe function?
Cowey and Green (1996)	12 (TLE)	Visuospatial motor task, letter span	Compared to FLE and HC, TLE patients were unimpaired on dual-task performance	No
	12 (FLE)			
	12 (HC)			
Duzel et al. (1996)	20 (TLE)	Corsi block tapping, digit span	TMS over left temporal lobe induces recency effects in verbal working memory task	Yes
Grippo et al. (1996)	29 (TLE)	Delayed match-to-sample	Specific ERP abnormalities in memory impaired TLE related to reduced working memory capacity	Yes
	26 (HC)			
Owen et al. (1996)	32 (FLEx)	Matched verbal, visual and spatial	TLEx and AHx groups impaired on visual working memory compared to FLEx. Spatial working memory deficits evident in TLEx only at high demand	Yes
	41 (TLEx)			
	19 (AHx)			
	91 (HC)			
Krauss et al. (1997)	8 (TLE)	Verbal and visuospatial	Mesial temporal spikes decreased working memory performance in 6/8 patients	Yes
Abrahams et al. (1999)	47 (TLE)	Nine-box maze	Spatial working memory deficits in right TLE patients	Yes
Axmacher et al. (2007)	11 (TLE)	Delayed match-to-sample	iEEG revealed sustained MTL activity during multiple item maintenance in TLE. Confirmed by MTL fMRI activity in HC	Yes
	23 (HC)			
Axmacher et al. (2008)	13 (TLE)	Delayed match-to-sample	MTL and inferior temporal lobe receive increasing top-down control as working memory load increase	Yes
	23 (HC)			
Axmacher et al. (2009)	8 (TLE)	iEEG activation patterns	Working memory related hippocampal deactivation interferes with long-term memory formation	Yes
	19 (HC)			
Campo et al. (2009)	9 (LHS)	MEG activity during verbal task	Reduced ipsilateral and increased contralateral MTL activity in TLE related to impaired performance	Yes
	10 (HC)			
Cashdollar et al. (2009)	6 (BHS)	MEG activity during spatial working memory	Hippocampus dependent networks critical for spatial WM. BHS but not LHS group showed reduced performance	Yes
	6 (LHS)			
	8 (HC)			
Wagner et al. (2009)	96 (TLE)	Matched verbal and visual supraspan tasks	Material specific deficits in working memory. Left TLE showed relatively more verbal deficits, right TLE showed relatively more visuospatial deficits	Yes
	30 (HC)			
Black et al. (2010)	207 (TLE)	Working memory index of WAIS-R	Earlier age of TLE onset predicts poorer outcome	Yes
	216 (PES)			
Vlooswijk et al. (2011)	36 (Cryp)	Delayed match-to-sample fMRI	Reduced prefrontal connectivity in patients compared to controls	Yes
	21 (HC)			

LHS: left hippocampal sclerosis; BHS: bilateral hippocampal sclerosis; TLE: temporal lobe epilepsy; FLE: frontal lobe epilepsy; PES: psychogenic non-epileptic seizures; FLEx; frontal lobe excision; TLEx: temporal lobe excision; AHx: selective amgdalohippocampectomy; Cryp: cryptogenic focal epilepsy; HC: healthy controls; ERP: event-related potential; TMS: transcranial magnetic stimulation; MEG: magnetoencephalography; iEEG; intracranial electroencephalogram; WM; working memory.

**Table 3:** Studies assessing pre- vs. postoperative change of frontal lobe function in TLE.

Author (yr)	*n*. (group)	Assessment	Postop assessment timecourse	Results
Hermann et al. (1988)	37 (TLE)	WCST	6 months	57% impaired preop. Fewer perseverative errors following surgery
	6 (PGE)			
Frisk and Milner (1990)	29 (TLE)	Word span	5 days and 2 weeks	Equal to HC preop; transient deficit at 5 days postop; restored at 2 weeks. Extent of excision of no significance
	14 (HC)			
Trenerry and Jack (1994)	34 (Left TLE)	WCST	4–5 months	No sig. difference in performance from before to after surgery
	34 (Right TLE)			
Hermann and Seidenberg, 1995	74 (TLE)	WCST	6 months	Postoperative improvements in performance
Martin et al. (1999)	15 (Left MTLE)	WCST, TMTb	Not reported	Executive function was not impaired preop and no sig. change postop for both groups
	40 (Left TLE + )			
Martin et al. (2000a)	174 (TLE)	WCST, VF TMTb	6–12 months	No change in WCST and TMTb scores, however VF sig. improved following surgery
Martin et al. (2000b)	89 (TLE)	WCST	6–12 months	No change postop. Seizure frequency outcome unrelated to WCST performance
Kim et al. (2007)	85 (MTLE)	WCST	1 year	56% MTLE impaired preop. Postop decline related to better preop performance
	34 (ncTLE)			

TLE: temporal lobe epilepsy; MTLE: mesial temporal lobe epilepsy; TLE+: temporal lobe epilepsy with coexisting temporal developmental malformation; ncTLE: neocortical temporal lobe epilepsy; PGE: primary generalised epielpsy; HC: healthy controls; WCST: Wisconsin card sorting task; MWCST: modified WCST; VF: verbal fluency; TMTb: Trail-Making-Test part B.

## References

[bib0005] Abrahams S., Morris R.G., Polkey C.E., Jarosz J.M., Cox T.C., Graves M., Pickering A. (1999). Hippocampal involvement in spatial and working memory, a structural MRI analysis of patients with unilateral mesial temporal lobe sclerosis. Brain Cogn..

[bib0010] Allegri R.F., Drake M., Thomson A. (1999). Neuropsychological findings in patients with middle temporal lobe epilepsy. Rev. Neurol..

[bib0015] Axmacher N., Elger C.E., Fell J. (2009). Working memory-related hippocampal deactivation interferes with long-term memory formation. J. Neurosci..

[bib0020] Axmacher N., Mormann F., Fernandez G., Cohen M.X., Elger C.E., Fell J. (2007). Sustained neural activity patterns during working memory in the human medial temporal lobe. J. Neurosci..

[bib0025] Axmacher N., Schmitz D.P., Wagner T., Elger C.E., Fell J. (2008). Interactions between medial temporal lobe, prefrontal cortex, and inferior temporal regions during visual working memory, a combined intracranial EEG and functional magnetic resonance imaging study. J. Neurosci..

[bib0030] Baddeley A. (2000). The episodic buffer, a new component of working memory?. Trends Cogn. Sci..

[bib0035] Bechara A., Damasio A.R., Damasio H., Anderson S.W. (1994). Insensitivity to future consequences following damage to human prefrontal cortex. Cognition.

[bib0040] Berg E.A. (1948). A simple objective technique for measuring flexibility in thinking. J. Gen. Psychol..

[bib0045] Black L.C., Schefft B.K., Howe S.R., Szaflarski J.P., Yeh H.S., Privitera M.D. (2010). The effect of seizures on working memory and executive functioning performance. Epilepsy Behav..

[bib0050] Cahn-Weiner D.A., Wittenberg D., McDonald C. (2009). Everyday cognition in temporal lobe and frontal lobe epilepsy. Epileptic Disord..

[bib0055] Campo P., Maestu F., Garcia-Morales I., Gil-Nagel A., Strange B., Morales M., Ortix T. (2009). Modulation of medial temporal lobe activity in epilepsy patients with hippocampal sclerosis during verbal working memory. J. Int. Neuropsychol. Soc..

[bib0060] Campo P., Maestu F., Ortiz T., Capilla A., Fernandez S., Fernandez A. (2005). Is medial temporal lobe activation specific for encoding long-term memories?. Neuroimage.

[bib0065] Cashdollar N., Malecki U., Rugg-Gunn F.J., Duncan J.S., Lavie N., Duzel E. (2009). Hippocampus-dependent and -independent theta-networks of active maintenance. Proc. Natl. Acad. Sci. U.S.A..

[bib0070] Cave C.B., Squire L.R. (1992). Intact verbal and nonverbal short-term memory following damage to the human hippocampus. Hippocampus.

[bib0075] Corcoran R., Upton D. (1993). A role for the hippocampus in card sorting?. Cortex.

[bib0080] Cowey C.M., Green S. (1996). The hippocampus, a working memory structure? The effect of hippocampal sclerosis on working memory. Memory.

[bib0085] Dabbs K., Jones J., Seidenberg M., Hermann B. (2009). Neuroanatomical correlates of cognitive phenotypes in temporal lobe epilepsy. Epilepsy Behav..

[bib0350] Devinsky O. (2005). The myth of the silent cortex and the morbidity of epileptogenic tissue: implications for temporal lobectomy. Epilepsy Behav..

[bib0090] Drake M., Allegri R.F., Thomson A. (2000). Executive cognitive alteration of prefrontal type in patients with mesial temporal lobe epilepsy. Med. B. Aires.

[bib0095] Duzel E., Hufnagel A., Helmstaedter C., Elger C. (1996). Verbal working memory components can be selectively influenced by transcranial magnetic stimulation in patients with left temporal lobe epilepsy. Neuropsychologia.

[bib0100] Ehlhardt L.A., Sohlberg M.M., Kennedy M., Coelho C., Ylvisaker M., Turkstra L., Yorkston K. (2008). Evidence-based practice guidelines for instructing individuals with neurogenic memory impairments: what have we learned in the past 20 years?. Neuropsychol. Rehabil..

[bib0105] Elger C.E., Helmstaedter C., Kurthen M. (2004). Chronic epilepsy and cognition. Lancet Neurol..

[bib0110] Frisk V., Milner B. (1990). The relationship of working memory to the immediate recall of stories following unilateral temporal or frontal lobectomy. Neuropsychologia.

[bib0115] Garcia Espinosa A., Andrade Machado R., Borges Gonzalez S., Garcia Gonzalez M.E., Perez Montoto A., Toledo Sotomayor G. (2010). Wisconsin Card Sorting Test performance and impulsivity in patients with temporal lobe epilepsy, suicidal risk and suicide attempts. Epilepsy Behav..

[bib0120] Gevins A., Cutillo B. (1993). Spatiotemporal dynamics of component processes in human working memory. Electroencephal. Clin. Neurophysiol..

[bib0125] Gilbert S.J., Burgess P.W. (2008). Executive function. Curr. Biol..

[bib0130] Giovagnoli A.R. (2001). Relation of sorting impairment to hippocampal damage in temporal lobe epilepsy. Neuropsychologia.

[bib0135] Grippo A., Pelosi L., Mehta V., Blumhardt L.D. (1996). Working memory in temporal lobe epilepsy, an event-related potential study. Electroencephalogr. Clin. Neurophysiol..

[bib0140] Hanna-Pladdy B. (2007). Dysexecutive syndromes in neurologic disease. J. Neurol. Phys. Ther..

[bib0145] Heaton R.K. (1981). Wisconsin Card Sorting Test Manual.

[bib0150] Hermann B.P., Wyler A.R., Richey E.T. (1988). Wisconsin Card Sorting Test performance in patients with complex partial seizures of temporal-lobe origin. J. Clin. Exp. Neuropsychol..

[bib0155] Hermann B.P., Seidenberg M., Haltiner A., Wyler A.R. (1991). Mood state in unilateral temporal lobe epilepsy. Biol. Psychiatry.

[bib0160] Hermann B., Seidenberg M. (1995). Executive system dysfunction in temporal lobe epilepsy, effects of nociferous cortex versus hippocampal pathology. J. Clin. Exp. Neuropsychol..

[bib0165] Hermann B., Seidenberg M., Lee E.J., Chan F., Rutecki P. (2007). Cognitive phenotypes in temporal lobe epilepsy. J. Int. Neuropsychol. Soc..

[bib0170] Hermann B.P., Seidenberg M., Schoenfeld J., Davies K. (1997). Neuropsychological characteristics of the syndrome of mesial temporal lobe epilepsy. Arch. Neurol..

[bib0175] Horner M.D., Flashman L.A., Freides D., Epstein C.M., Bakay R.A.E. (1996). Temporal lobe epilepsy and performance on the Wisconsin Card Sorting Test. J. Clin. Exp. Neuropsychol..

[bib0180] Jokeit H., Seitz R.J., Markowitsch H.J., Neumann N., Witte O.W., Ebner A. (1997). Prefrontal asymmetric interictal glucose hypometabolism and cognitive impairment in patients with temporal lobe epilepsy. Brain.

[bib0185] Keeler J.F., Robbins T.W. (2011). Translating cognition from animals to humans. Biochem. Pharmacol..

[bib0190] Keller S.S., Baker G., Downes J.J., Roberts N. (2009). Quantitative MRI of the prefrontal cortex and executive function in patients with temporal lobe epilepsy. Epilepsy Behav..

[bib0195] Kim C.H., Lee S.A., Yoo H.J., Kang J.K., Lee J.K. (2007). Executive performance on the Wisconsin Card Sorting Test in mesial temporal lobe epilepsy. Eur. Neurol..

[bib0355] Kim S.Y., Lee H.W., Jung D.K., Suh C.K., Park S.P. (2006). Cognitive ffects of low-dose topiramate compared with oxcarbazepine in epilepsy patients. J. Clin. Neurol..

[bib0200] Krauss G.L., Summerfield M., Brandt J., Breiter S., Ruchkin D. (1997). Mesial temporal spikes interfere with working memory. Neurology.

[bib0205] Labudda K., Frigge K., Horstmann S., Aengenendt J., Woermann F.G., Ebner A., Markowitsch H., Brand J.M. (2009). Decision making in patients with temporal lobe epilepsy. Neuropsychologia.

[bib0210] Linden D.E. (2007). The working memory networks of the human brain. Neuroscientist.

[bib0215] Martin R., Dowler R., Gilliam F., Faught E., Morawetz R., Kuzniecky R. (1999). Cognitive consequences of coexisting temporal lobe developmental malformations and hippocampal sclerosis. Neurology.

[bib0220] Martin R.C., Sawrie S.M., Edwards R., Roth D.L., Faught E., Kuzniecky R.I., Morawetz R.B., Gilliam F.G. (2000). Investigation of executive function change following anterior temporal lobectomy, selective normalization of verbal fluency. Neuropsychology.

[bib0225] Martin R.C., Sawrie S.M., Gilliam F.G., Palmer C.A., Faught E., Morawetz R.B., Kuzniecky R.I. (2000). Wisconsin Card Sorting performance in patients with temporal lobe epilepsy, clinical and neuroanatomical correlates. Epilepsia.

[bib0230] McDonald C.R., Ahmadi M.E., Hagler D.J., Tecoma E.S., Iragui V., Garapetian L., Dale A.M., Halgren E. (2008). Diffusion tensor imaging correlates of memory and language impairments in temporal lobe epilepsy. Neurology.

[bib0235] McDonald C.R., Delis D.C., Norman M.A., Tecoma E.S., Iragui-Madozi V.I. (2005). Is impairment in set-shifting specific to frontal-lobe dysfunction? Evidence from patients with frontal-lobe or temporal-lobe epilepsy. J. Int. Neuropsychol. Soc..

[bib0240] Mueller S.G., Laxer K.D., Barakos J., Cheong I., Finlay D., Garcia P., Cardenas-Nicolson V., Weiner M.W. (2009). Involvement of the thalamocortical network in TLE with and without mesiotemporal sclerosis. Epilepsia.

[bib0245] Nelson H.E. (1976). A modified card sorting test sensitive to frontal lobe defects. Cortex.

[bib0250] Oddo S., Solis P., Consalvo D., Giagante B., Silva W., D‘Alessio L., Centurión E., Saidón P., Kochen S. (2003). Mesial temporal lobe epilepsy and hippocampal sclerosis, cognitive function assessment in Hispanic patients. Epilepsy Behav..

[bib0255] Owen A.M., McMillan K.M., Laird A.R., Bullmore E. (2005). N-back working memory paradigm, a meta-analysis of normative functional neuroimaging studies. Human Brain Mapp..

[bib0260] Owen A.M., Morris R.G., Sahakian B.J., Polkey C.E., Robbins T.W. (1996). Double dissociations of memory and executive functions in working memory tasks following frontal lobe excisions, temporal lobe excisions or amygdalo-hippocampectomy in man. Brain.

[bib0265] Oyegbile T.O., Dow C., Jones J., Bell B., Rutecki P., Sheth R., Seidenberg M., Hermann B.P. (2004). The nature and course of neuropsychological morbidity in chronic temporal lobe epilepsy. Neurology.

[bib0270] Penn D.C., Povinelli D.J. (2007). On the lack of evidence that non-human animals possess anything remotely resembling a ‘theory of mind’. Philos. Trans. Roy. Soc. Lond., Sect. B, Biol. Sci..

[bib0275] Ragland J.D., Yoon J., Minzenberg M.J., Carter C.S. (2007). Neuroimaging of cognitive disability in schizophrenia: search for a pathophysiological mechanism. Int. Rev. Psychiatry.

[bib0280] Ranganath C., Blumenfeld R.S. (2005). Doubts about double dissociations between short- and long-term memory. Trends Cogn. Sci..

[bib0285] Ranganath C., D’Esposito M. (2001). Medial temporal lobe activity associated with active maintenance of novel information. Neuron.

[bib0290] Schacher M., Winkler R., Grunwald T., Kraemer G., Kurthen M., Reed V., Jokeit H. (2006). Mesial temporal lobe epilepsy impairs advanced social cognition. Epilepsia.

[bib0360] Smith M.E., Gevins A., McEvoy L.K., Meador K.J., Ray P.G., Gilliam F. (2006). Distinct cognitive neurophysiologic profiles for lamotrogine and topiramate. Epilepsia.

[bib0295] Squire L.R., Stark C.E., Clark R.E. (2004). The medial temporal lobe. Ann. Rev. Neurosci..

[bib0300] Stern C.E., Sherman S.J., Kirchhoff B.A., Hasselmo M.E. (2001). Medial temporal and prefrontal contributions to working memory tasks with novel and familiar stimuli. Hippocampus.

[bib0365] Stopford C.L., Thompson J.C., Neary D., Richardson A.M., Snowden J.S. (2011). Working memory, attention, and executive function in Alzheimer's disease and frontotemporal dementia. Cortex.

[bib0310] Strauss E., Hunter M., Wada J. (1993). Wisconsin card sorting performance, effects of age of onset of damage and laterality of dysfunction. J. Clin. Exp. Neuropsychol..

[bib0315] Takaya S., Hanakawa T., Hashikawa K., Ikeda A., Sawamoto N., Nagamine K., Ishizu K., Fukuyama H. (2006). Prefrontal hypofunction in patients with intractable mesial temporal lobe epilepsy. Neurology.

[bib0320] Thompson P.J., Duncan J.S. (2005). Cognitive decline in severe intractable epilepsy. Epilepsia.

[bib0325] Trenerry M.R., Jack C.R. (1994). Wisconsin Card Sorting Test – performance before and after temporal lobectomy. J. Epilepsy.

[bib0330] Vlooswijk M.C., Jansen J.F., Jeukens C.R., Marian Majoie H.J., Hofman P.A., de Krom M.C., Aldenkamp A.P., Backes W.H. (2011). Memory processes and prefrontal network dysfunction in cryptogenic epilepsy. Epilepsia.

[bib0335] Wagner D.D., Sziklas V., Garver K.E., Jones-Gotman M. (2009). Material-specific lateralization of working memory in the medial temporal lobe. Neuropsychologia.

[bib0340] Wang X.Q., Lang S.Y., Lu H., Ma L., Mao Y.L., Yang F. (2007). Executive function impairment in patients with temporal lobe epilepsy, neuropsychological and diffusion-tensor imaging study. Zhonghua Yi Xue Za Zhi.

[bib0345] Zarahn E., Rakitin B., Abela D., Flynn J., Stern Y. (2005). Positive evidence against human hippocampal involvement in working memory maintenance of familiar stimuli. Cereb. Cortex.

